# Bioinformatics analysis of the immune cell infiltration characteristics and correlation with crucial diagnostic markers in pulmonary arterial hypertension

**DOI:** 10.1186/s12890-023-02584-4

**Published:** 2023-08-15

**Authors:** Guili Lian, Jingxian You, Weijun Lin, Gufeng Gao, Changsheng Xu, Huajun Wang, Li Luo

**Affiliations:** 1 Department of Geriatrics, The First Affiliated Hospital of Fujian Medical University, Chazhong Road 20, Taijiang District, Fuzhou, 350005 People’s Republic of China; 2 Fujian Hypertension Research Institute, The First Affiliated Hospital of Fujian Medical University, Fuzhou, 350005, People’s Republic of China; 3 Clinical Research Center for Geriatric Hypertension Disease of Fujian Province, The First Affiliated Hospital of Fujian Medical University, Fuzhou, 350005 People’s Republic of China; 4 Branch of National Clinical Research Center for Aging and Medicine, The First Affiliated Hospital of Fujian Medical University, Fujian Province, Fuzhou, 350005 People’s Republic of China; 5 Department of Geriatrics, National Regional Medical Center, Binhai Campus of the First Affiliated Hospital, Fujian Medical University, Fuzhou, 350212 People’s Republic of China

**Keywords:** Pulmonary arterial hypertension, Bioinformatics analysis, Immune infiltration, Hub gene, LTBP1

## Abstract

**Background:**

Pulmonary arterial hypertension (PAH) is a pathophysiological syndrome, characterized by pulmonary vascular remodeling. Immunity and inflammation are progressively recognized properties of PAH, which are crucial for the initiation and maintenance of pulmonary vascular remodeling. This study explored immune cell infiltration characteristics and potential biomarkers of PAH using comprehensive bioinformatics analysis.

**Methods:**

Microarray data of GSE117261, GSE113439 and GSE53408 datasets were downloaded from Gene Expression Omnibus database. The differentially expressed genes (DEGs) were identified in GSE117261 dataset. The proportions of infiltrated immune cells were evaluated by CIBERSORT algorithm. Feature genes of PAH were selected by least absolute shrinkage and selection operator (LASSO) regression analysis and validated by fivefold cross-validation, random forest and logistic regression. The GSE113439 and GSE53408 datasets were used as validation sets and logistic regression and receiver operating characteristic (ROC) curve analysis were performed to evaluate the prediction value of PAH. The PAH-associated module was identified by weighted gene association network analysis (WGCNA). The intersection of genes in the modules screened and DEGs was used to construct protein–protein interaction (PPI) network and the core genes were selected. After the intersection of feature genes and core genes, the hub genes were identified. The correlation between hub genes and immune cell infiltration was analyzed by Pearson correlation analysis. The expression level of LTBP1 in the lungs of monocrotaline-induced PAH rats was determined by Western blotting. The localization of LTBP1 and CD4 in lungs of PAH was assayed by immunofluorescence.

**Results:**

A total of 419 DEGs were identified, including 223 upregulated genes and 196 downregulated genes. Functional enrichment analysis revealed that a significant enrichment in inflammation, immune response, and transforming growth factor β (TGFβ) signaling pathway. CIBERSORT analysis showed that ten significantly different types of immune cells were identified between PAH and control. Resting memory CD4^+^ T cells, CD8^+^ T cells, γδ T cells, M1 macrophages, and resting mast cells in the lungs of PAH patients were significantly higher than control. Seventeen feature genes were identified by LASSO regression for PAH prediction. WGCNA identified 15 co-expression modules. PPI network was constructed and 100 core genes were obtained. Complement C3b/C4b receptor 1 (CR1), thioredoxin reductase 1 (TXNRD1), latent TGFβ binding protein 1 (LTBP1), and toll-like receptor 1 (TLR1) were identified as hub genes and LTBP1 has the highest diagnostic efficacy for PAH (AUC = 0.968). Pearson correlation analysis showed that LTBP1 was positively correlated with resting memory CD4^+^ T cells, but negatively correlated with monocytes and neutrophils. Western blotting showed that the protein level of LTBP1 was increased in the lungs of monocrotaline-induced PAH rats. Immunofluorescence of lung tissues from rats with PAH showed increased expression of LTBP1 in pulmonary arteries as compared to control and LTBP1 was partly colocalized with CD4^+^ cells in the lungs.

**Conclusion:**

LTBP1 was correlated with immune cell infiltration and identified as the critical diagnostic maker for PAH.

**Supplementary Information:**

The online version contains supplementary material available at 10.1186/s12890-023-02584-4.

## Introduction

Pulmonary hypertension (PH) is a pathophysiological syndrome, which is characterized by increased pulmonary vascular resistance (PVR) and pulmonary vascular remodeling, leading to heart failure, and eventually death [1]. Pulmonary arterial hypertension (PAH) is Group 1 PH defined as a mean pulmonary arterial pressure > 20 mmHg at rest with a capillary wedge pressure ≤ 15 mmHg and PVR ≥ 3 Wood units [2, 3]. PAH-targeted therapies, including drugs targeting at nitric oxide pathway, endothelin receptors, prostaglandin receptors, thromboxane receptors, and phosphodiesterase inhibitors, improve symptoms and hemodynamics of PAH [4]. However, the mortality of PAH patients remains high with a five-year survival rate is about 60% [5]. Thus, it’s urgent to identify new diagnostic biomarkers and treatment targets for PAH.

The pathological mechanism of PAH is complex and has not been fully understood. Immunity and inflammation are progressively recognized properties of PAH. A large amount of evidence has demonstrated that inflammation and immunity play vital roles in the initiation and maintenance of pulmonary vascular remodeling in PAH [6–9]. An accumulation of macrophages, B and T cells, mast cells, and dendritic cells (DCs) was observed in the lungs or around the pulmonary vessels of PAH [9–13]. Infiltration of macrophages in the lungs and dysregulated RIPK3-mediated necroptosis and its triggered toll-like receptor (TLR) and NOD-like receptor (NLR) pathways were observed in the pathogenesis of PAH in our previous studies [7, 8]. However, the immune mechanisms of PAH have not been well explored. Thus, it is a need to evaluate the proportions of immune cells and screen critical hub genes related to immune cells in the PAH using systematic and integrated bioinformatics methods.

With extensive use of gene profiles from Gene Expression Ominous (GEO), bioinformatics analysis can be used to identify potential biomarkers and mechanisms for PAH. Previous studies have identified potential biomarkers and abnormal immune cell infiltration between PAH and normal control in different datasets downloaded for the GEO database using different bioinformatics methods [14, 15]. Our previous studies have identified PNISR and HNRNPH1 may be potential biomarkers to provide a better diagnosis of PAH [16]. In this study, the GSE117261 were downloaded to screen differentially expressed genes (DEGs) between PAH and control and further explore their potential biological functions via enrichment analysis. LASSO, random forest (RF), and logistic regression (LR) were used to identify and validate the biomarkers of PAH in GSE113439 and GSE53408 datasets. WGCNA and PPI network analysis were performed to identify core genes related to PAH. We identified the critical hub genes that could distinguish PAH between control by the intersection of feature genes and core genes. Immune cell infiltration was investigated in PAH by CIBERSORT. The correlation between immune cells and hub genes was evaluated by Pearson correlation analysis. This study aimed to explore immune cell infiltration characteristics and potential biomarkers of PAH using comprehensive bioinformatics analysis.

## Materials and methods

### GEO dataset collection and data preprocessing

**Fig. 1:**
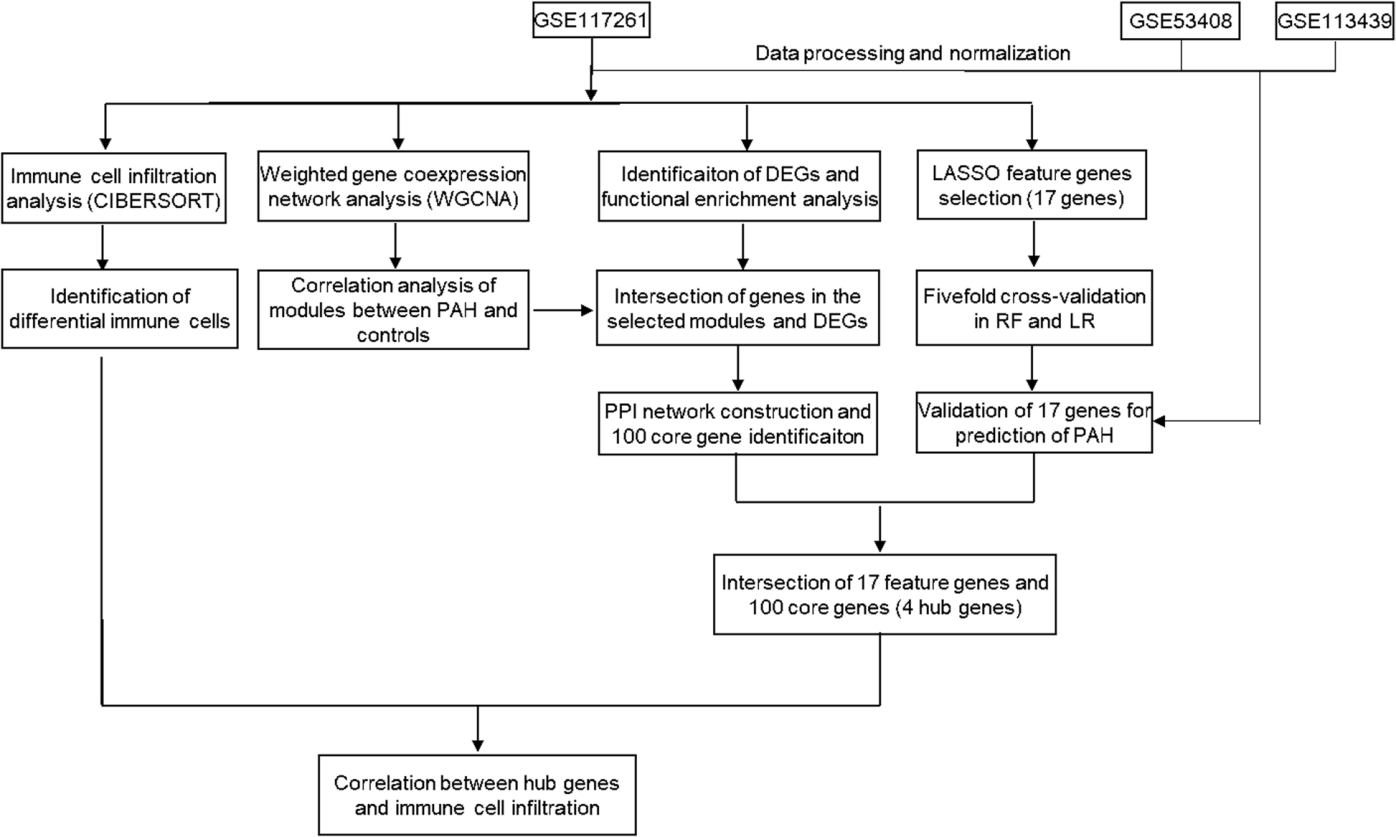
The flowchart of the bioinformatics analysis. GSE117261, GSE113439 and GSE53408 datasets were downloaded from the GEO database. After pre-processing and normalization of the data, the differentially expressed genes (DEGs) were identified in GSE117261 and the functional enrichment analyses of Gene Ontology and KEGG were performed. GSEA was conducted to investigate the potential biological pathways using the entire gene set. The immune landscape in the dataset was determined by the CIBERSORT algorithm. Lasso regression analysis was performed to identify 17 feature genes, and fivefold cross-validation was performed using RF and LR in GSE117261. ROC curve of 17 feature genes was performed to construct PAH prediction model in GSE53408 and GSE113439. WGCNA was performed to identify the modules associated with PAH. The intersection of genes in the modules screened and DEGs were used to construct PPI network and identification of the core genes. After the intersection of 17 feature genes and 100 core genes, four hub genes were identified. Pearson correlation analysis was performed to analyze the correlation between the hub genes and immune cell infiltration

Gene expression profiles in patients with PAH were downloaded from the GEO database (https://www.ncbi.nlm.nih.gov/geoprofiles/). Three datasets were selected, including GSE117261 [16, 17], GSE113439 [18] and GSE53408 [19–21]. GSE117261 (58 patients with PAH and 25 control) was used as a training set, GSE113439 (15 patients with PAH and 11 control) and GSE53408 (12 patients with PAH and 11 control) were used as validation sets, and all three datasets were based on GLP6244 platform (Affymetrix Human Gene 1.0ST Array). Before and after batch correction, the gene expression matrix of three dataset visualized by box plot and PCA plot. The flow chart of data processing and analysis was shown in Fig. 1.

### Identification of differentially expressed genes (DEGs) and functional enrichment analysis

The DEGs between PAH patients and control in the GSE117261 dataset were selected by the limma2 package. The ggplot2 package was used to generate the volcano plot of DEGs, and the pheatmap package was used to draw the heat map of DEGs. |log2 fold change (FC)|> 0.5, adjusted *P* value < 0.05 was set as a cutoff for this selection. GO and KEGG enrichment analyses (http://www.genome.jp/kegg/) [22–24] for DEGs were performed by R package clusterProfiler with the filter with adjusted *P* value < 0.05. The gene set enrichment analysis (GSEA) was also performed by clusterProfiler with filter condition as false discovery rate (FDR) < 0.25 and *P* value < 0.05.

### Evaluation of immune cell infiltration

CIBERSORT is a method of analyzing the composition and abundance of immune cells in a mixed-cell population using gene expression data based on the principle of linear support vector regression. The gene expression matrix was uploaded to CIBERSORT and derived a matrix of 22 types of immune cells. CIBERSORT *P* < 0.05 was used to filter the samples. The distribution of 21 types of immune cells in the samples was calculated and displayed in a bar plot and the difference in immune cells between the two groups was displayed in a violin plot using the ggplot2 package in R language. PCA results of immune cell infiltration matrix in GSE117261 were obtained using R package stats. A correlation heatmap was constructed to visualize the correlation of hub genes with 22 types of immune cells using a corrplot package.

### Predictive model construction

The predictive power of gene expression of lungs between PAH patients and control was assessed by three machine learning algorithms, least absolute shrinkage and selection operator (LASSO) regression analysis, random forest (RF) and logistic regression (LR). The feature genes were screened by five-fold cross-validation and the GSE117261 dataset was split into a training set and a test set with a 4:1 ratio. LASSO classifier was performed on the training set. The performance of the prediction model of PAH was evaluated by RF and LR in the test set. The optimal prediction method generated by RF was selected and validated in the external datasets GSE113439 and GSE53408 and the area under the receiver operating characteristic (ROC) curve (AUC) was calculated after cross-validation.

### Weighted Gene Association Network Analysis (WGCNA)

The WGCNA package in R was used to construct the WGCNA gene expression matrix in the GSE117261 dataset. Firstly, the correlation coefficient between genes was calculated, and the weighted value of the correlation coefficient was used to make the connection between genes in the scale-free network. Secondly, a hierarchical clustering tree was constructed according to the correlation coefficients between genes. Different dendrogram branches represent different genes and different colors represented different modules. Thirdly, the module significance (MS) was calculated and used to measure the correlation of traits with different modules and record the genes in each module. The correlation between sample traits and genes was explored by gene significance (GS). The modules with the strongest correlation were selected separately, and the GS and module membership (MM) values were visualized in scatter plots.

### PPI network construction and core genes identification

The upregulated DEGs and downregulated DEGs were intersected with the genes in the dark olive green and dark green modules identified by WGCNA, respectively, and the obtained genes were used to construct a protein–protein interaction (PPI) network through STRING (https://string-db.org/). The PPI network was further visualized by Cytoscape. The CytoHubba Cytospace plugin was used to identify core genes by the MCC algorithm.

### Correlation analysis between immune cells and hub genes

Pearson correlation analysis was used to analyze the relationship between different immune cells and feature genes in R software. The plots were visualized by the “ggplot2” package and *P* < 0.05 was considered statistically significant.

### Animals

The SD rats weighing 150 ± 20 g were purchased from Shanghai SLAC Laboratory Animal CO, Ltd.(Certificate No. SCXK 2017–0005). The rats were housed in the Animal Center of Fujian Medical University and received food and water ad libitum*.* After one week of adaptation, the rats were subcutaneously injected with 20 mg/kg monocrotaline (MCT, Sigma) twice, at a 7-day interval or normal saline as previously described [8, 25]. The rats were anesthetized with 50 mg/kg sodium pentobarbital at week 4 after MCT injection. The right ventricular catheterization was used to determine to mean pulmonary artery pressure (mPAP) by PE50 tube and Powerlab system (ADInstruments, Australia). The rats were sacrificed after hemodynamics measurement. The right ventricular hypertrophy index (RVHI) was calculated as right ventricule/left ventricule + septum. The lung morphology was evaluated by HE staining and observed with Nikon Eclipse E200 microscope. The pulmonary remodeling index (WT% and WA%) was analyzed. All procedures have been conducted in accordance with the ARRIVE guidelines and were approved by the Animal Welfare and Ethics Committee of Fujian Medical University (No. FJMU IACUC 2021–0378).

### Cell experiments

Rats were deeply anesthetized with 50 mg/kg sodium pentobarbital and euthanized by cervical dislocation. Primary PASMCs were prepared from pulmonary arteries using a previously described protocol [25]. The cells were maintained in Dulbecco’s modified Eagle’s medium/F12 (DMEM/F12, BasalMedia, China) supplemented with 10% fetal bovine serum (FBS, Excell, South America) and 1% penicillin–streptomycin (Servicebio, China) at 37 °C in an atmosphere of 5% CO2. Cells were starved in 0.5% FBS supplemented medium for 24 h and then treated with 20 ng/ml PDGF-BB (Peprotech, Rocky Hill, NJ, United States) for 48 h. The PASMCs from generation 3 to 5 were used in the present experiments.

### Western blot

For the analysis of total proteins, lung tissues or PASMCs were lysed by RIPA buffer with a cocktail and PMSF and homogenated by tissue homogenator (Wuhan Service Biotechnology). The lysates were centrifuged for 15 min at 13000 g, 4℃. The concentration of total protein was determined by the BCA assay kit (Beijing Biyuntian Biotechnology). The lysates were eluted with 2*SDS buffer and boiled for 10 min at 100℃. The samples were loaded and separated on 10% SDS/PAGE gel, and then transferred onto polyvinylidene difluoride membranes (PVDF) (Millipore, USA). The membranes were blocked in 5% non-fat milk and then incubated with specific primary antibodies (anti β-actin Mouse antibody [GB12001, Wuhan Servicebio Technology, China], and anti-LTBP1 antibody [ab78294, Abcam, UK]) at a dilution of 1:1000 at 4 °C overnight. After being incubated with HRP-conjugated secondary antibodies, the blots were visualized using the ECL system (New Cell and Molecular Biotech Co, Ltd, China), captured by iBright 1500 (Invitrogen by Thermo Fisher Scientific) and analyzed by Image J software.

### Immunohistochemistry (IHC) and immunofluorescence (IF)

For IHC, the left lung tissues were fixed in 10% neutral formalin for 24 h and dehydrated by ethanol from 30 to 100% concentration. The samples were immersed in xylene and embedded in paraffin. Then the sections (5 μm) were cut, deparaffinized, and rehydrated. After antigen retrieval with EDTA (pH9.0), the slides were incubated with 3% H2O2 for 10 min and blocked with 10% goat serum for 30 min. The section was incubated with LTBP1 antibody (1:200 ab78294, Abcam) overnight at 4℃. After being washed 3 times in PBS, the slides were incubated with biotinylated secondary antibody for 1 h and then incubated with HRP-labeled streptavidin for another 30 min at room temperature. 20ul DAB to 1 ml of DAB substrate was mixed by swirling, applied to tissue, and incubated for 10 min. Hematoxylin was used to stain the nucleus. The section was covered by mounting medium and observed by microscope (Eclipse E200, Nikon).

For IF, the sections were blocked with 10% nonfat milk for 30 min after antigen retrieval and incubated with LTBP1 antibody (1:200, ab78294) and CD4 antibody (1:50, 67786–1-Ig, Proteintech, China) overnight at 4℃. After being washed 3 times in PBS, the slides were incubated with goat anti-rabbit IgG (Alexa Fluor 488) and goat anti-mouse IgG (Alexa Fluor 594) for 1 h at room temperature. The section was covered by mounting medium and observed by inverted fluorescence microscope (Eclipse Ts2, Nikon).

### Statistical analysis

All statistical tests and visual analyses were performed in R software (version 3.6.1). The R package "ggpurbr" is used to calculate statistical parameters in visual box plots. Continuous variables were expressed as mean ± SD, and the differences between two groups were compared using Student’s *t*-test. The Wilcox-t test was used to compare the differences in immune cell infiltration and gene expression between PAH and control groups. The sensitivity and specificity of feature genes to distinguish PAH from control were assessed using a ROC curve. All statistical tests were two-sided and *P* < 0.05 was considered significantly different.

## Results

### Data processing

**Fig. 2:**
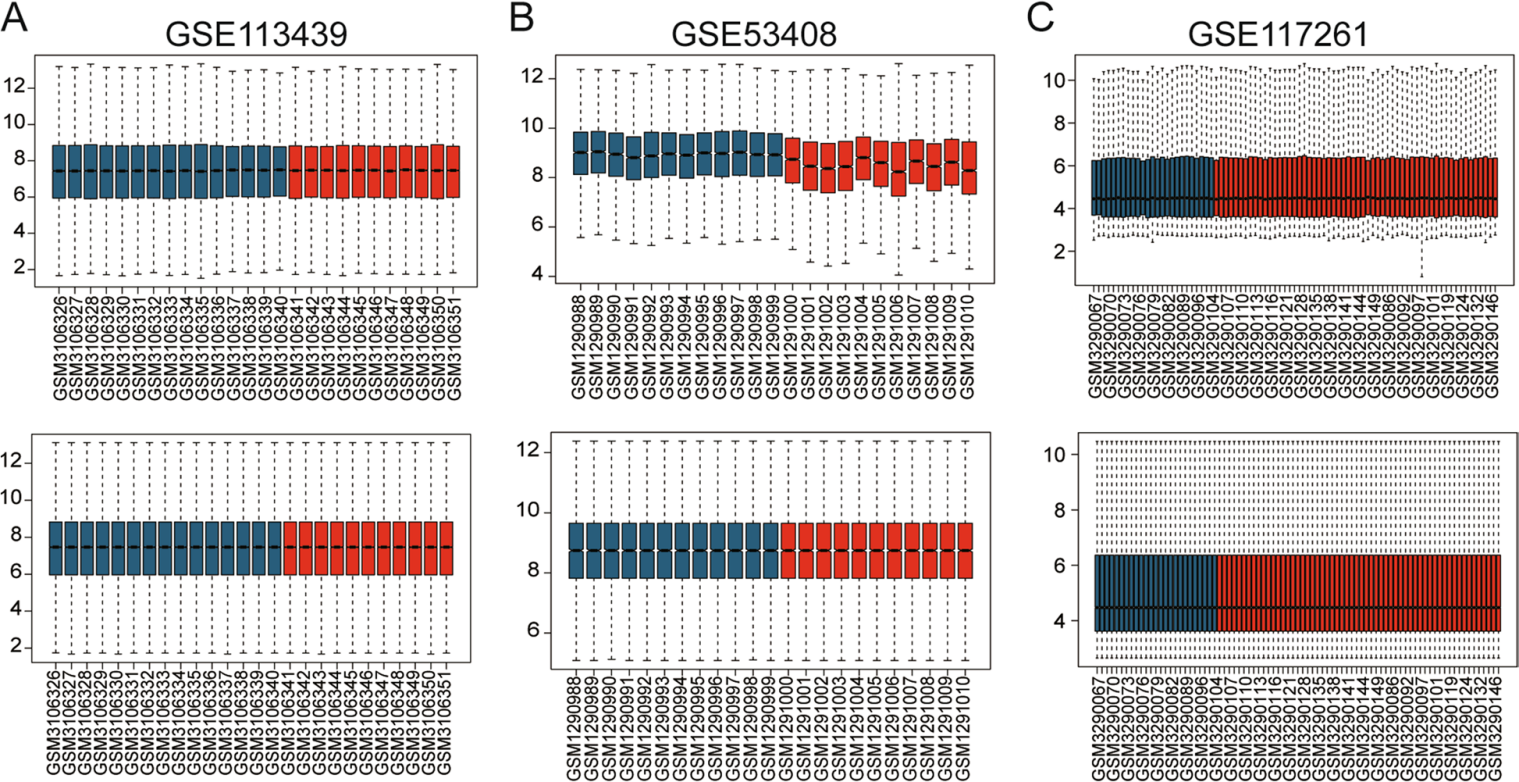
The box diagram of the gene expression matrix before and after normalization. **A** GSE113439 expression profile before and after normalization; **B** GSE113439 expression profile before and after normalization; **C** GSE53408 expression profile before and after normalization. The red color represents PAH lung tissue samples, and the blue color represents normal lung tissue samples

Three microarray raw datasets, GSE117261, GSE53408, and GSE113439 were selected for the present study. All expression values in GSE117261, GSE53408, and GSE113439 datasets before and after normalization were presented as box diagrams in Fig. 2 and the PCA charts were shown in Figure S1.

### Identification of DEGs and functional enrichment analyses

**Fig. 3:**
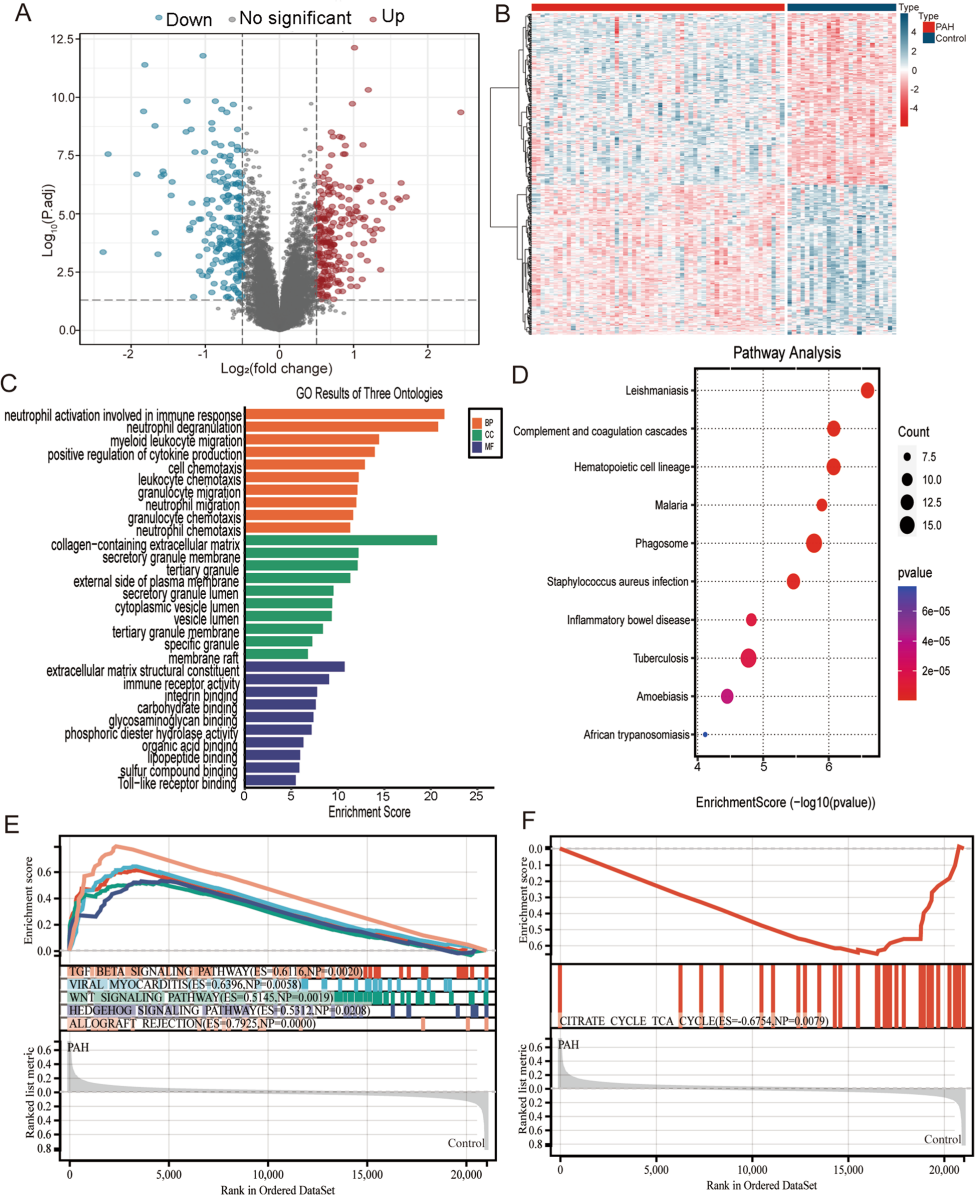
Screening and functional enrichment analysis of DEGs. **A** The volcano plot shows DEGs between PAH and normal control. The blue dots represent downregulated DEGs, the red dots represent upregulated DEGs and the grey dots represent the genes that were not significantly changed. **B** The DEGs were visualized by the heatmap with red color for upregulation and blue color for downregulation. The enrichment analysis of DEGs includes GO functional analysis (**C**) and KEGG pathway enrichment (**D**); **E** GSEA plot showed that top five enriched KEGG pathways were positively correlated to PAH; **F** GSEA plot showed that TCA cycle was negatively correlated to PAH

The normalized GSE117261 was used to identify DEGs between PAH and normal control lung tissues. A total of 419 DEGs were obtained, including 223 upregulated genes and 196 downregulated genes. The detailed information on the DEGs was listed in Table S1. The DEGs were visualized by the volcano map and heat map (Fig. 3A, B). GO function, and KEGG pathway analyses were performed for the DEGs. The GO annotations of DEGs consisted of three parts, including biological process (BP), cellular component (CC), and molecular function (MF), which were used to analyze the functional enrichment of DEGs. The DEGs were mainly related to neutrophil activation involved in immune response, neutrophil degranulation, myeloid leukocyte migration, positive regulation of cytokine production and cell chemotaxis in GO function analysis (Fig. 3C). KEGG enrichment analysis showed that the DEGs were enriched in inflammatory disease and complement and coagulation cascades (Fig. 3D). GSEA was performed to discover crucial biological pathways and potential mechanism using gene expression profiles. According to the ranking and enrichment scores, the top six pathways were shown in Fig. 3E and F. TGFβ signaling pathway, viral myocarditis, Wnt signaling pathway, Hedgehog signaling pathway and allograft rejection were positively correlated with PAH, whereas the tricarboxylic acid (TCA) cycle was negatively correlated with PAH.

### Immune infiltration analysis

**Fig. 4:**
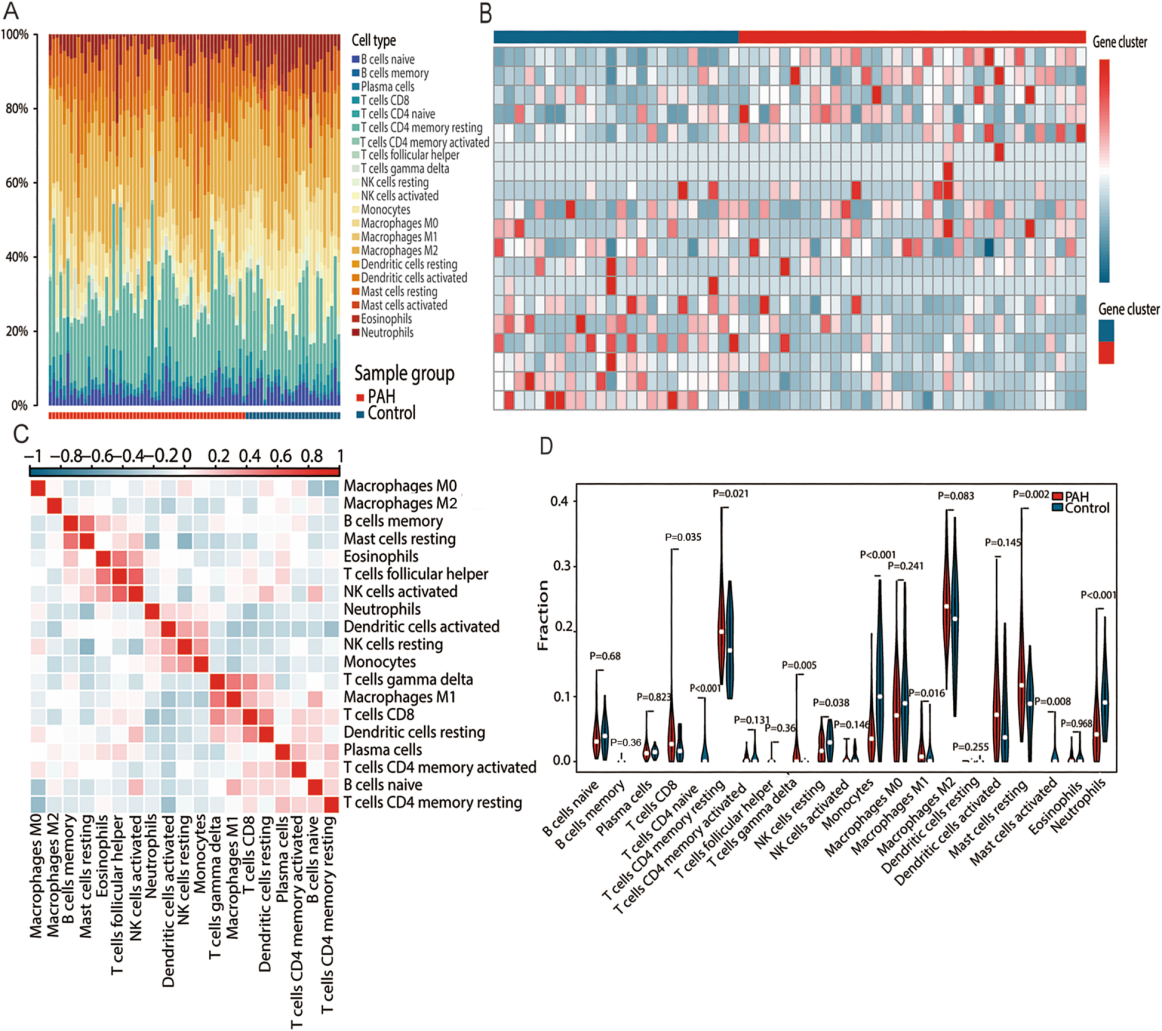
The difference in immune cell infiltration between PAH and control. **A** The proportions of 21 immune infiltrating cells showed in the histogram; **B** The clustering of 19 types of immune cells in a heatmap; **C** A correlation heatmap of the proportions of 19 immune cells; **D** The violin plot showing the differences in 21 types of immune cells between PAH and normal control

The fractions of 21 types of immune cells in each sample of GSE117261 were presented in a histogram (Fig. 4A). The color represents the percentage of different immune cells in each sample, and the sum is 1. Figure 4B showed that all samples were divided into two groups by clustering the abundance of 19 types of immune cells. The PCA plot demonstrated that the patterns of immune cells in PAH and control were different (Figure S2). Pearson correlation analysis revealed that the monocytes were negatively correlated with resting mast cells and resting CD4^+^ T cells, but positively correlated with neutrophils, and resting NK cells (Fig. 4C). The differences in fractions of immune cells between PAH and control lung tissues were presented in a violin plot (Fig. 4D). The results showed CD8^+^ T cells, resting memory CD4^+^ T cells, γδ T cells, M1 macrophages and resting mast cells in lungs of PAH patients were significantly higher than control, whereas CD4^+^ T naive cells, resting NK cells, activated mast cells, monocytes, and neutrophils were lower.

### Construction of prediction model for PAH and identification of feature genes

**Fig. 5:**
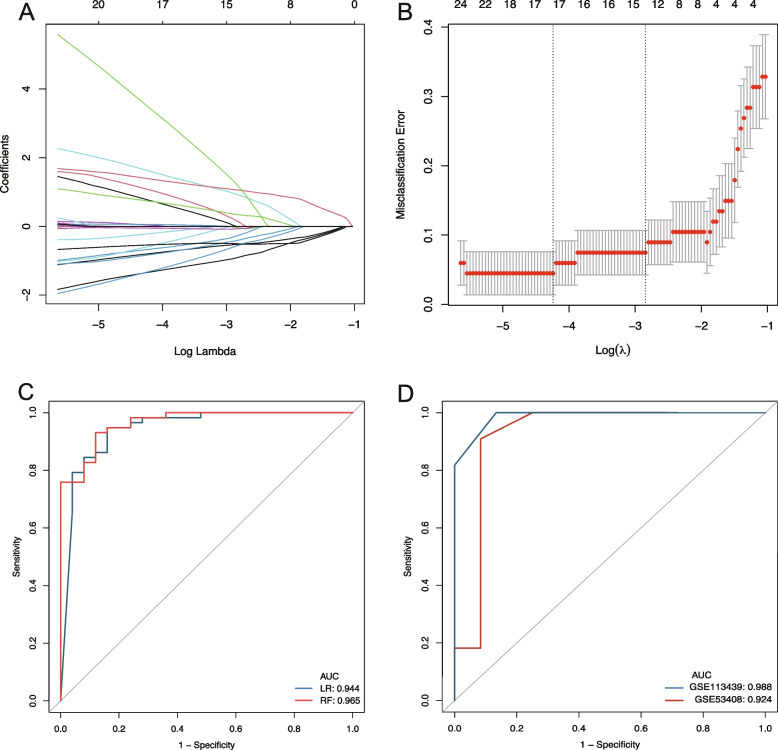
Establishment of PAH prediction model. **A**, **B** LASSO coefficient spectrum for identification of feature genes of PAH and control samples from GSE117261. **C** The result of ROC analysis of internal validation datasets in GSE117261 using LR and RF; **D** Externally validated ROC analysis in the GSE113439 and GSE53408 datasets. LASSO: least absolute shrinkage and selection operator; RF: random forest; LR: logistic regression

A dimension reduction was performed by the LASSO algorithm in the training set and 17 feature genes were identified in the prediction model of PAH (Fig. 5A, B; Table S2). The mRNA profiles of the 17 genes in the dataset GSE53408 and GSE113439 were visualized by heatmap in Figure S2A and B respectively. RF and LR were used to the evaluate effectiveness of the perdition model in the test set. The predictive value of the mRNA expression profiles was evaluated by the area under the receiver operating characteristic (ROC) curve (AUC). The results showed that the 17 feature genes could predict PAH. The AUC calculated by RF was 0.965, and the calculated by LR was 0.944 (Fig. 5C). The RF was selected for external validation, and it was found that the feature genes discriminated PAH from normal control in GSE113439 (AUC = 0.988) or GSE53408 (AUC = 0.924) (Fig. 5D). Besides, the heatmap and ROC of 17 genes, including NUCB2, ZNF724P, CYLD, HIVEP1, TXNRD1, UTP3, UEVLD, ZRANB3, TLR1, ACPP, CR1, LTBP1, SULT1B1, NKD1, METRNL, HYI and RAPPES2 in the GSE3439 and GSE53408 datasets were shown in Figure S3C-F.

### WGCNA

**Fig. 6:**
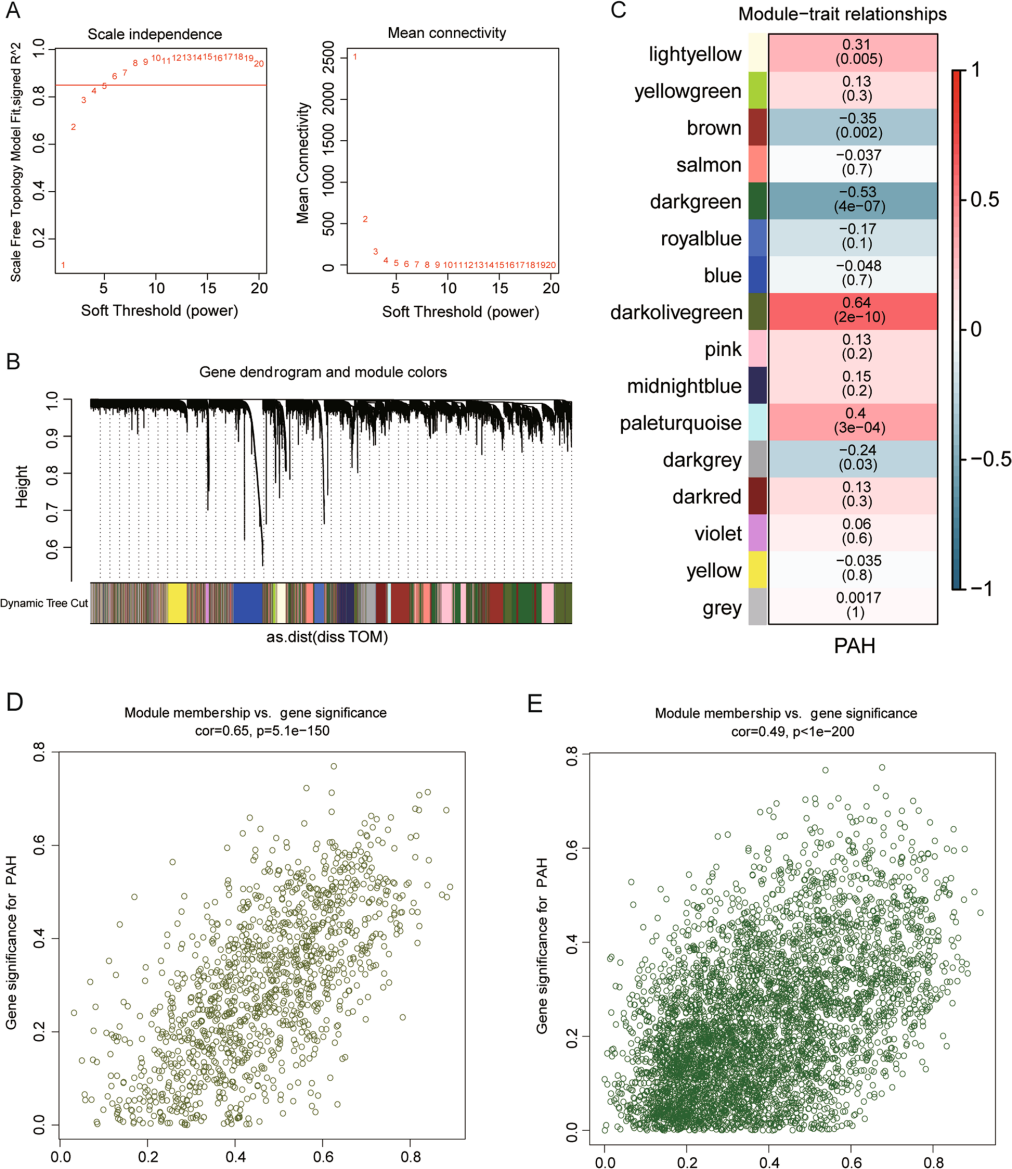
Construction of WGCNA network. **A** Analysis of the scale-free index and mean connectivity for various soft-threshold powers (1 ~ 20). **B** Dendrogram of 15 modules of genes with different colors. **C** Correlation heatmap showing 15 modules of different colors associated with PAH. The scatter plots showing gene distribution within the dark olive green module (**D**) and the dark green modules (**E**), respectively

To classify and analyze the impact of the different gene expression profiles in the samples of the GSE117261 dataset, the weight co-expression network was constructed using the WGCNA software package. The soft threshold (power) was determined as five based on the scale-free fit index and mean connectivity in Fig. 6A. A total of fifteen co-expression modules were identified and displayed in Fig. 6B. The relationship between modules and traits was evaluated using Pearson’s correlation analysis. As shown in Fig. 6C, the two most correlated modules (dark olive green module and dark green module) were identified, and the results showed that the dark olive green module was positively correlated with PAH (R2 = 0.64, *P* < 0.001), and the dark green module was negatively correlated with PAH (R2 = -0.53, *P* < 0.001). The GS and MM values of all genes in the dark olive green module and the dark green module were shown in the scatter plots (Fig. 6D and E). 1,242 genes in the dark olive green (Table S3) and 4,543 genes (Table S4) in the dark green module were obtained for subsequent analysis.

### Construction of PPI network and identification of core genes

**Fig. 7:**
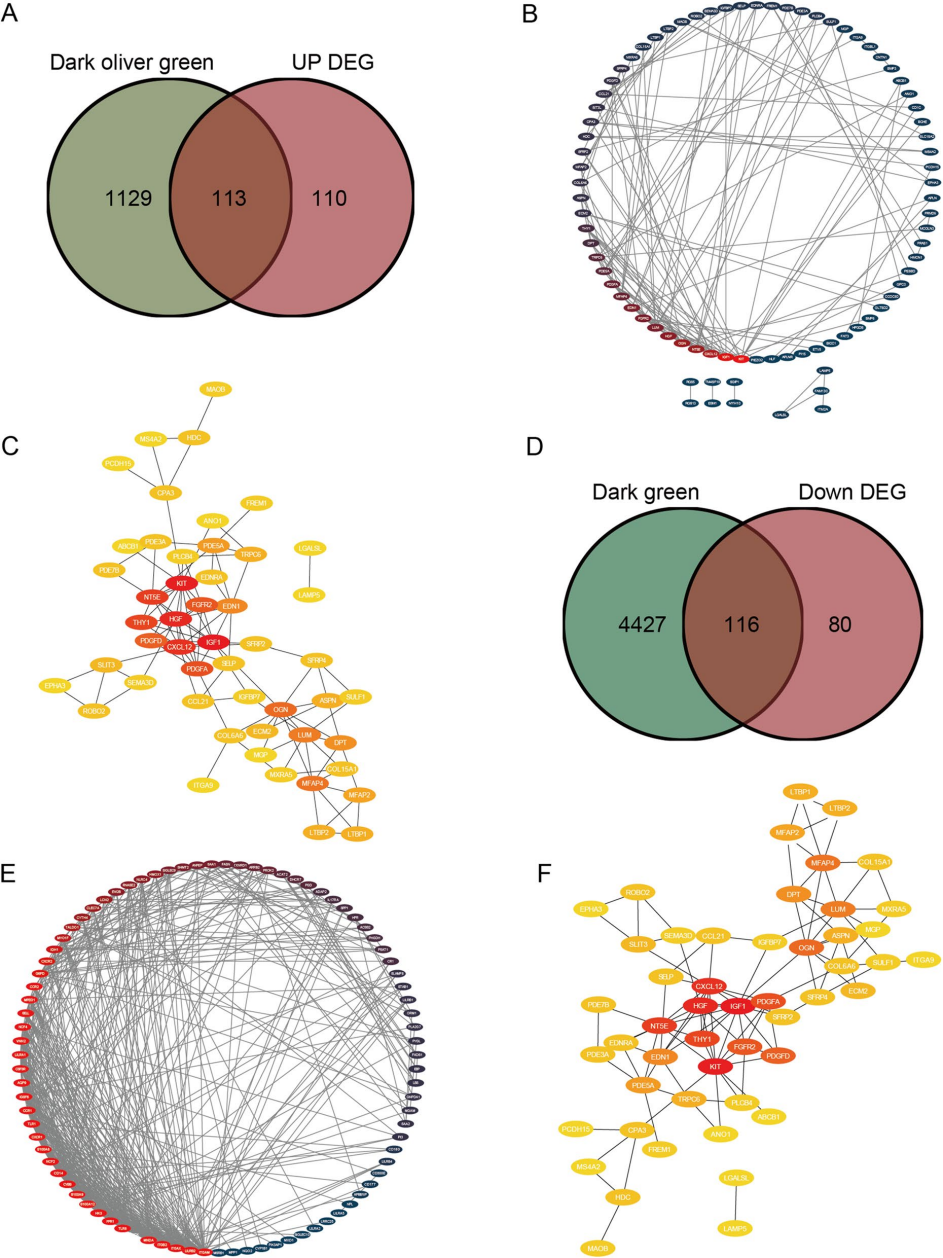
Construction of a PPI network. **A** The Venn diagram of the intersection of the upregulated DEGs and the genes in the dark olive module. **B** The protein interaction network of genes in the intersection. The red color represents high weight; the blue color represents low weight. **C** Screening of 50 upregulated hub genes. **D** The Venn diagram of the intersection of the downregulated DEGs and the genes in dark green modules. **E** Protein interaction network of genes in the intersection. The red color represents high weight and the blue color represents low weight. **F** Screening of 50 downregulated hub genes

To further screen the core genes, the 223 upregulated DEGs were intersected with 1,242 genes in the dark olive green and 196 downregulated DEGs were intersected with 4,543 genes in the dark green module, then 113 upregulated DEGs and 116 downregulated genes were obtained (Fig. 7A, D). Then, the two groups of genes were respectively constructed for the PPI network using the STRING database. The upregulated DEGs network included 82 nodes and 141 edges (Fig. 7B) and the downregulated DEGs network included 87 nodes and 479 edges (Fig. 7E). The two networks were screened for core genes through the MCC algorithm in CytoHubba, and the top 50 core genes were selected respectively (Fig. 7C, F).

### The hub genes identification

**Fig. 8:**
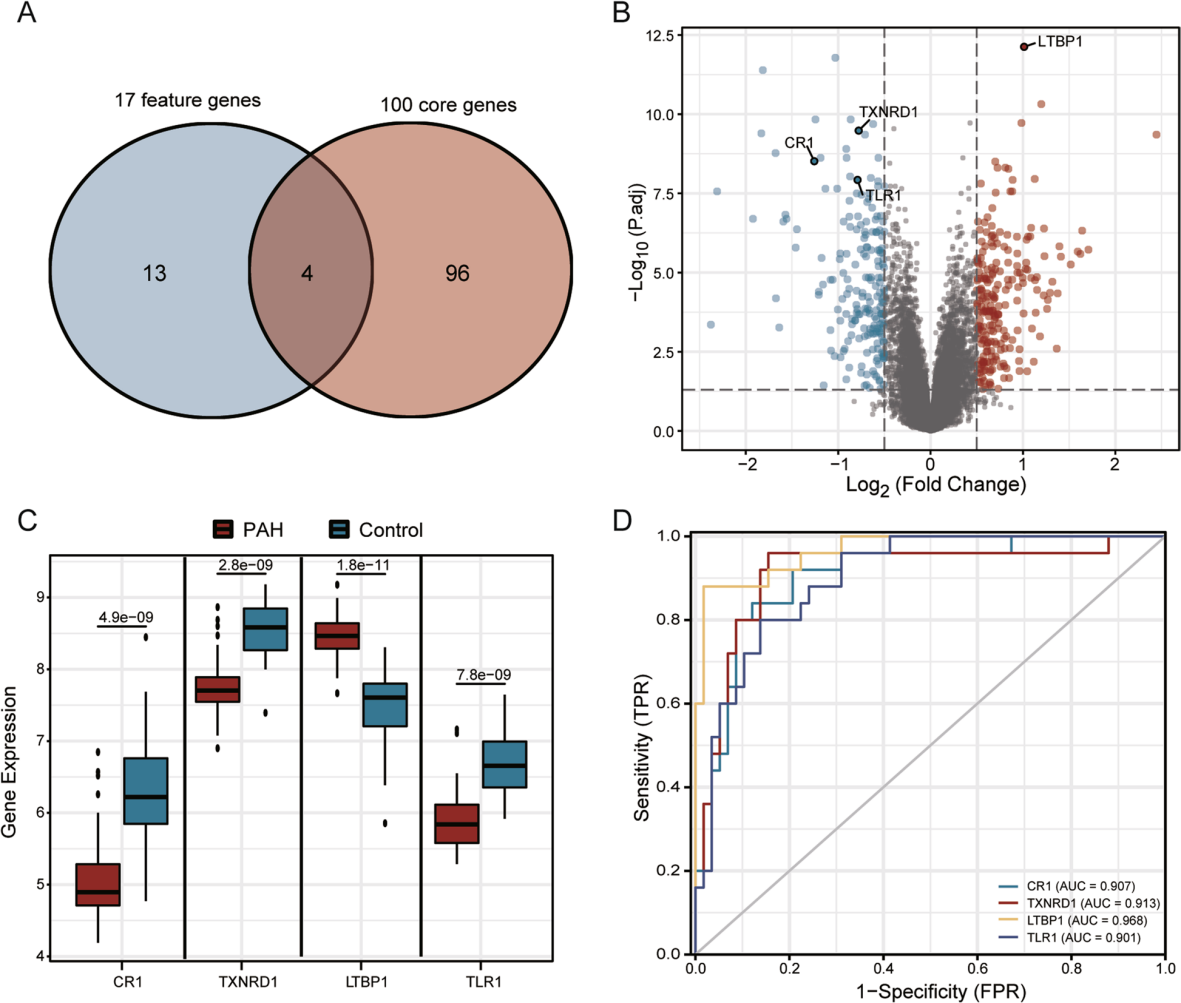
Identification of the hub genes. **A** The Venn diagram of the intersection of 17 feature genes and 100 core genes; **B** 4 hub genes marked in the volcano plot of all genes. **C** The difference of the four key genes between PAH and control. **D** ROC curves of 4 hub genes as independent diagnostic indicators

The 100 core genes were further intersected with the 17 feature genes identified by LASSO regression, then 4 hub genes complement C3b/C4b receptor 1 (CR1), thioredoxin reductase 1 (TXNRD1), latent TGFβ binding protein 1 (LTBP1) and toll-like receptor 1 (TLR1) were obtained (Fig. 8A) and marked from the volcano plot (Fig. 8B). The difference in mRNA expression of CR1, LTBP1, TXNRD1, and TLR1 between PAH and the control was shown in a box plot (Fig. 8C). LTBP1 was increased in PAH, while CR1, TXNRD1 and TLR1 were downregulated in comparison with control in the GSE117261 dataset. The four genes were used as independent indicators to test the diagnostic efficacy and displayed by the ROC curve (Fig. 8D). The results revealed that LTBP1 (AUC = 0.968), TXNRD1 (AUC = 0.913), CR1 (AUC = 0.907), and TLR1 (AUC = 0.901) have good diagnostic efficacy as independent indicators for PAH. Among them, LTBP1 has the highest diagnostic efficacy.

### Correlation between hub genes and infiltrating immune cells

**Fig. 9:**
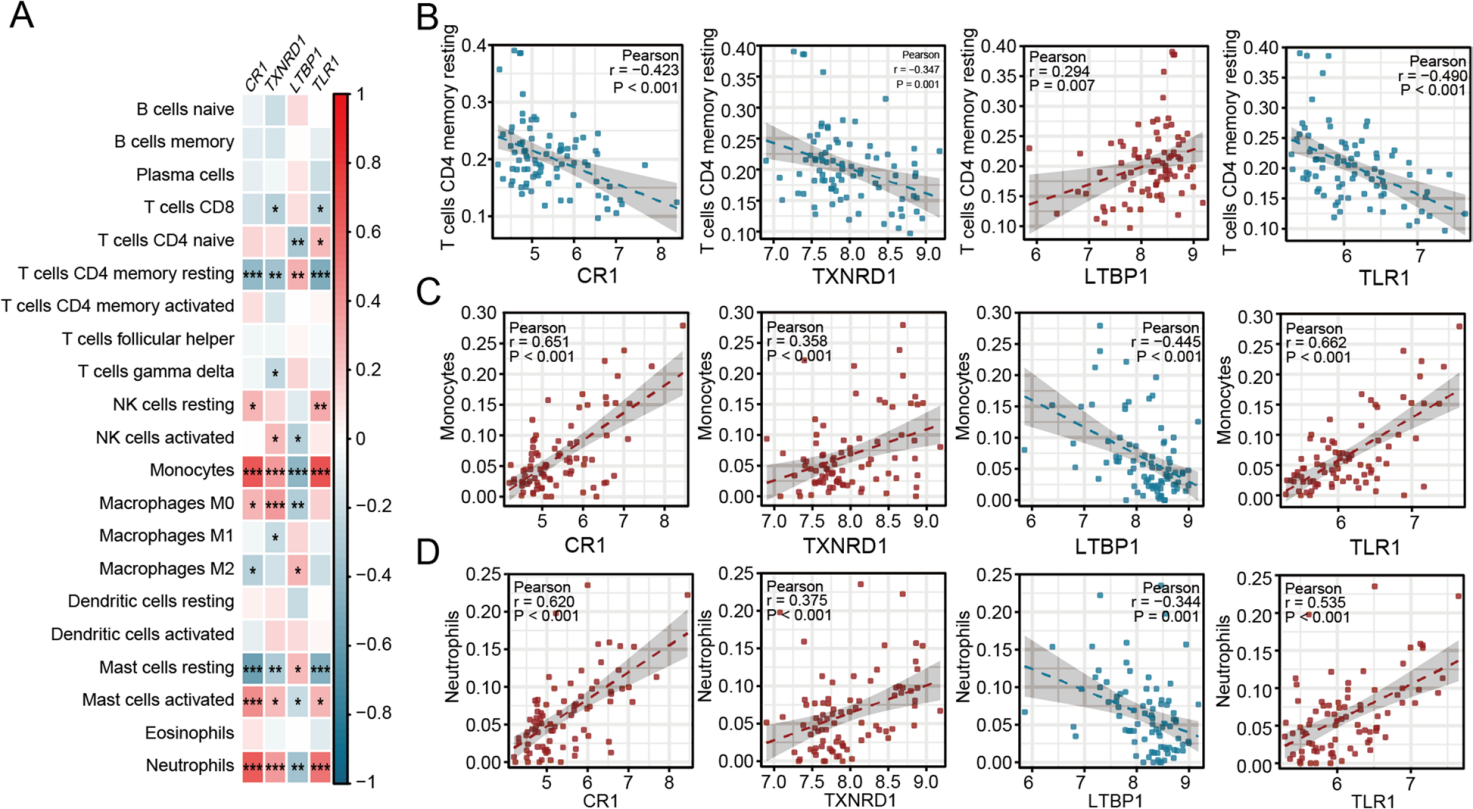
Correlation analysis between hub genes and immune cells. **A** A heatmap of the correlation between the hub genes and immune cells. The red color represents a positive correlation, and the blue color represents a negative correlation, * *P* < 0.05; ** *P* < 0.01; ***P < 0.001. **B** The relationship between resting memory CD4.^+^ T cells with CR1, TXNRD1, LTBP1 and TLR1; **C** The correlation of monocytes to CR1, TXNRD1, LTBP1 and TLR1; **D** The correlation of neutrophils to CR1, TXNRD1, LTBP1 and TLR1

To explore the roles of hub genes in PAH, Pearson correlation analysis was performed between immune infiltrating cells and the hub genes in GSE117261 dataset. The result was displayed in a heatmap in Fig. 9A and showed that CR1, TXNRD1, and TLR1 had a similar relationship with immune cells in contrast to LTBP1. Especially, the four hub genes strongly correlated with resting memory CD4^+^ T cells, monocytes and neutrophils. The scatter plots showed that CR1 (monocytes *r* = 0.651; neutrophils *r* = 0.620; all *P* < 0.001), TXNRD1 (monocytes *r* = 0.358; neutrophils *r* = 0.375; all *P* < 0.001) and TLR1 (monocytes* r* = 0.662; neutrophils* r* = 0.535; all *P* < 0.001) were positively correlated with monocytes and neutrophils, repcetively, whereas negatively correlated with resting memory CD4^+^ T cells (CR1 *r* = -0.423, *P* < 0.001; TXNRD1 *r* = -0.347, *P* = 0.001; TLR1 *r* = -0.490, *P* < 0.001). LTBP1 was positively correlated with memory resting CD4^+^ T cells (*r* = 0.294, *P* = 0.007), but negatively correlated with monocytes (*r* = -0.358,* P* < 0.001) and neutrophils (*r* = -0.344, *P* = 0.001) (Fig. 9B, C, D).

### LTBP1 expression was increased in the PAH animal model and the (PDGF-BB)-induced PASMCs

**Fig. 10:**
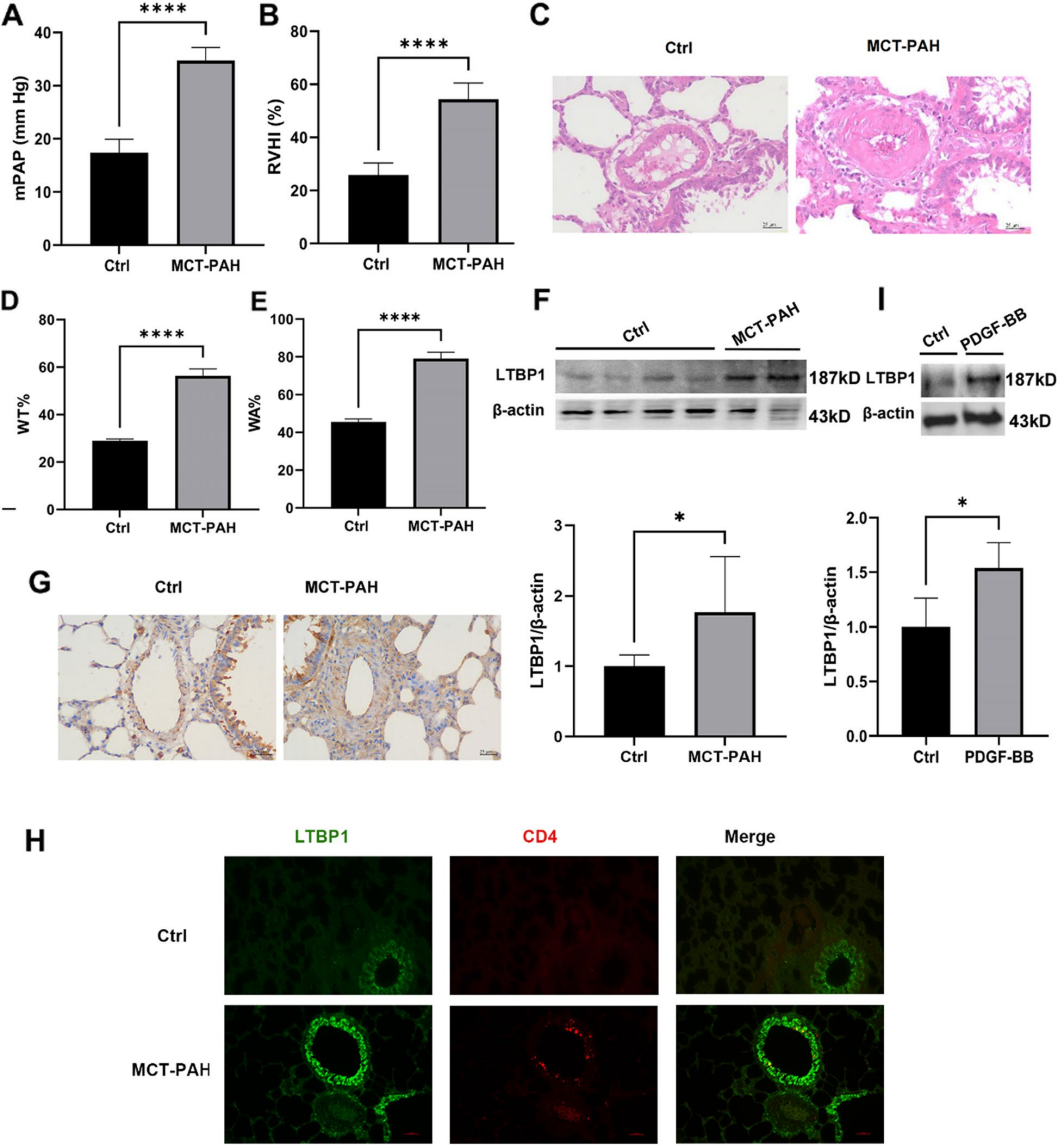
LTBP1 was upregulated in monocrotaline (MCT)-induced PAH and (PDGF-BB)-induced PASMCs. **A**, **B** mPAP and RVHI were increased in rats of MCT-PAH. **C** HE staining of the lung in MCT-induced PAH rats and control (Scale bar = 25 μ m). **D**, **E** WT% and WA % of pulmonary arteries were calculated from HE staining. **F** Western blotting and quantification of LTBP1 and β-actin in the lungs of (MCT)-induced PAH (*n* = 9) and control (*n* = 8). **G** Immunohistochemistry analysis of LTBP1 in lungs, Scale bar = 50 μm. **H** Immunofluorescence of lung tissues with LTBP1 (green) and CD4 (red); Scale bar = 50 μm; **I** Western blot and quantification of LTBP1 and β-actin in the (PDGF-BB)-induced PASMCs and control, *n* = 4. Data represent the mean ± SD and Student *t*-test was used to compare the two groups. ****: *P* < 0.0001 *vs* Ctrl; *: *P* < 0.05 *vs* Ctrl. MCT: monocrotaline; Ctrl: control; MCT-PAH: MCT-induced pulmonary arterial hypertension

Based on that LTBP1 has the highest diagnostic efficacy for PAH and was consistently increased in the GSE117261, GSE113439 and GSE53408 datasets, we confirmed the expression level of LTBP1 in MCT-induced PAH rat model. Compared with the control, the mPAP and RVHI were increased in PAH rats (Fig. 10A, B). HE staining demonstrated that pulmonary arterial remodeling and immune cell infiltration in the lungs of PAH rats (Fig. 10C). The pulmonary remodeling indices (WT% and WA%) were also increased in PAH rats (Fig. 10D, E). The level of LTBP1 protein was significantly increased in the lungs of MCT-induced PAH rats by Western blotting (Fig. 10F). IHC analysis showed that LTBP1 expression was also enhanced in the pulmonary arteries and lungs of PAH rats (Fig. 10G). Immunofluorescence of lung tissues from rats with PAH showed increased expression of LTBP1 in pulmonary arteries as compared to control and LTBP1 was partly localized with CD4^+^ cells in the lungs (Fig. 10H). We also validated that the expression of LTBP1 protein was significantly increased in the (PDGF-BB)-induced PASMCs by Western blotting (Fig. 10I).

## Discussion

PAH is life-threatening disease characterized by pulmonary vascular remodeling, the underlying mechanism and immune infiltration involved in the remodeling of pulmonary arteries are not fully understood. Increasing evidence has shown that inflammation and immune cells play important roles in the remodeling of pulmonary arteries and the development of PAH [7, 9, 25] Therefore, it is a need to explore the immune pathogenesis of PAH and search for novel effective biomarkers. In the present study, we used integrated bioinformatics methods to investigate the role of immune cell infiltration in the PAH and identify effective diagnostic biomarkers for PAH.

This study explored the immune cell infiltration characteristics and correlation with crucial diagnostic markers in pulmonary arterial hypertension by bioinformatics analysis. KEGG, GO and GSEA analysis revealed that a significantly enrichment in inflammation, immune response, and transformed growth factor β (TGFβ) signaling pathway. We found that a significant difference in immune cell infiltration between PAH and normal control with accumulation of memory resting CD4^+^ T cells, CD8^+^ T cells, γδ T cells, M1 macrophages and resting mast cells, but decreased monocytes, neutrophils, naive CD4^+^ T cells, resting NK cells, and activated mast cells in PAH patients. The four crucial hub genes for diagnosis of PAH were identified by LASSO regression, WGCNA and PPI network analysis. LTBP1 was increased, while CR1, TXNRD1 and TLR1 were decreased in PAH in comparison with control. CR1, TXNRD1 and TLR1 were positively correlated with monocytes and neutrophils, whereas negatively correlated with memory resting CD4^+^ T cells. LTBP1 was negatively correlated with monocytes and neutrophils, but positively correlated with memory resting CD4^+^ T cells. This study has found the immune cell infiltration characteristics and correlation with crucial diagnostic markers in PAH.

Human and animal PAH studies have demonstrated various inflammatory mediators were increased in both PAH patients and animal models of PAH [15]. Consistent with previous findings, the DEGs were mainly enriched in immune response, such as neutrophil activation involved in immune response, neutrophil degranulation, myeloid leukocyte migration, positive regulation of cytokine production and cell chemotaxis. KEGG enrichment analysis showed that the DEGs were enriched in inflammatory disease and complement and coagulation cascades. These finding suggested that immune response and inflammation participates in the pathogenesis of PAH [26]. GSEA was performed to discover crucial biological pathways and potential mechanism using gene expression profiles and the results demonstrated that TGFβ signaling pathway were positively correlated with PAH. TGFβ signaling pathway was overactivated and leads to excessive proliferation and resistance of apoptosis [27, 28]. TGF-β1 is an essential regulator of extracellular matrix (ECM) deposition, promoting fibrosis and inflammation [29]. These studies confirmed that the immune response and inflammation play important roles in PAH.

Accumulation of multiple immune cells, such as T cells, B cells, macrophages, mast cells and DCs were also observed around the remodeled pulmonary vessels or near plexiform lesions in animal models and PAH patients [7, 9]. A single-cell study of two rat PH models revealed that a significant increase in the cell fractions of interstitial macrophage in MCT-induced rats and alveolar macrophage in hypoxia/su5416 induced rats [13]. The comprehensive infiltration characteristics of immune cells in the lungs of PAH were investigated in our study and ten significantly different types of immune cells between PAH and control were identified by CIBERSORT algorithm. Similar to previous studies, the proportions of CD8^+^ T cells, resting CD4^+^ T cells, γδ T cells, M1 macrophages and resting mast cells were significantly increased in lungs of PAH patients. However naive CD4^+^ T cells, resting NK cells, activated mast cells, monocytes and neutrophils were significantly decreased in PAH patients compared to control [15, 30]. Differently, a previous study observed significantly higher proportion of naive CD4^+^ T cells and central memory T cells but lower proportions of cytotoxic T cells, exhausted T cells, type 17 T helper cells, effector memory T cells, NK T cells, NK cells, γδT cells, and CD8^+^ T cells in PAH by another tool ImmuCellAI (Immune Cell Abundance Identifier) [31]. The inconsistency of the results may be due to use of different datasets and analysis methods. Thus, the exploration of novel biomarkers related to immune cell infiltration via an integrated bioinformatics analysis shows promise for the treatment of PAH.

We identified 17 feature genes for the prediction of PAH by LASSO regression. The two most significantly related modules were found by WGCNA and the genes in the two modules were intersected with DEGs. The genes in the intersection were used to construct PPI network and then 100 core genes were screened. Four hub genes CR1, TXNRD1, TLR1 and LTBP1 were identified in the insection of 17 feature genes and 100 core genes. CR1, TXNRD1 and TLR1 were decreased in PAH, whereas LTBP1 was increased in PAH. CR1/CD35, a member of the receptors of complement activation (RCA) family, plays important roles in the immune response and complement cascade. At present, CR1 has not yet been reported in PAH. In our study, CR1 was downregulated in PAH and positively correlated with monocytes and neutrophils, but negatively correlated with memory resting CD4^+^ T cells, which provided some evidence for future investigation of the mechanism of CR1-associated immune cell infiltration in PAH. TXNRD1, a member of the thioredoxin (Trx) system that catalyzes Trx1 reduction, plays a vital role in redox homeostasis [32]. In our study, it was shown that TXNRD1 mRNA was significantly decreased in human PAH lung samples by bioinformatics, which is consistent with previous reports [6, 33]. TXNRD1 was also downregulated in the serum of PAH patients and lungs of MCT-induced PAH rats [6, 34]. Knockdown of TXNRD1 inhibited platelet-derived growth factor (PDGF)-BB promoted pulmonary arterial smooth muscle cells (PASMCs) proliferation and migration. We also found TXNRD1 was positively correlated with monocytes and neutrophils, whereas negatively correlated with memory resting CD4^+^ T cells. TLR1 plays a key role in the innate immune system. As a member of the TLRs family, TLR1 also plays a fundamental role in pathogen recognition and activation of innate immunity. Our previous study demonstrated that TLR2 was upregulated in lung of MCT-induced PAH rats and dysregulated TLR and NLR pathways was identified in the progression of pulmonary vascular remodeling in PAH [7]. However TLR1 was decreased in the lung of human PAH patients and has a positive correlation with monocytes and neutrophils in the study. TLR1 may contribute to PAH via regulation of monocytes and neutrophils-related immune response and inflammation.

LTBP1, belonging to the family of LTBPs, targets latent complexes of TGFβ to ECM and regulation TGFβ activation. Leppäranta et al. [35] reported that LTBP1 was significantly upregulated inidiopathic pulmonary fibrosis (IPF) patient lungs and modulated TGF-β availability and activation in different pulmonary compartments in the fibrotic lung. TGFβ signaling pathway was overactivated in PAH and lead to excessive proliferation, the resistance of apoptosis and ECM deposition [29, 30]. ECM deposition in remodeling pulmonary arteries is thought to be a key characteristic of PAH. Our study demonstrated LTBP1 was increased in human PAH samples by bioinformatics analysis. According to the ROC curve, the values of the AUC of LTBP1 was 0.968 with a highest ability to discriminate PAH from the control. We also confirmed that LTBP1 was upregulated in lung and pulmonary arteries of PAH rats. The correlation coefficient between LTBP1 and CD4^+^ T cells *r* = 0.294, but the difference was significant. IF results showed that LTBP1 was partly colocalized with CD4 in lungs of PAH. These results indicate that LTBP1 was correlated to immune cells and may regulate immune cell function and thus play an important role in the process of PAH. Since LTBP1 is one component of ECM and regulates TGFβ signaling pathway and related to immunity and inflammation, it was considered as a candidate biomarkers of PAH. Furthermore, further studies are needed to investigate the role of LTBP1 in the development of PAH in vivo and in vitro.

## Conclusion

In conclusion, LTBP1 is upregulated and correlated to immune infiltration in PAH, identified as a new critical biomarker for PAH. Our study suggests that LTBP1 is involved in the development of PAH and serves as a potential diagnostic and therapeutic target for PAH.

### Supplementary Information

**Additional file 1: Figure S1.** PCA plots of three datasets before and after batch correction. **Figure S2.** The PCA plot of immune cells between PAH and control in GSE117261 dataset. **Figure S3.** Heatmap of 17 feature genes in GSE113439 and GSE53408 datasets. **Figure S4.** The ROC of 17 genes in GSE117261.  **Table S1.** Details of the DEGs in the dataset GSE117261. **Table S2.** Identification of seventeen characteristic genes of PAH using LASSO regression algorithm. **Table S3.** The genes in the dark olive green module by WGCNA. **Table S4.** The genes in the dark green module by WGCNA. 

## Data Availability

The mRNA expression dataset used in our study was downloaded from the Gene Expression Omnibus (GEO) under accession number GSE117261, GSE53408, GSE113439 (https://www.ncbi.nlm.nih.gov/geo/query/acc.cgi?acc=GSE, accessed on 5 June 2021).
