# Supplementary material for: Bioinformatics analysis of the immune cell infiltration characteristics and correlation with crucial diagnostic markers in pulmonary arterial hypertension
Source: BMC Pulm Med. 2023 Aug 15;23:300. doi: 10.1186/s12890-023-02584-4 (PMC10428559; doi:10.1186/s12890-023-02584-4)
Supplement: Supplementary file 1 — Additional file 1: Figure S1. PCA plots of three datasets before and after batch correction. Figure S2. The PCA plot of immune cells between PAH and control in GSE117261 dataset. Figure S3. Heatmap of 17 feature genes in GSE113439 and GSE53408 datasets. Figure S4. The ROC of 17 genes in GSE117261. Table S1. Details of the DEGs in the dataset GSE117261. Table S2. Identification of seventeen characteristic genes of PAH using LASSO regression algorithm. Table S3. The genes in the dark olive green module by WGCNA. Table S4. The genes in the dark green module by WGCNA. [file 12890_2023_2584_MOESM1_ESM.zip › Supplementary material/Figure S1-S4.docx]

Supplementary material


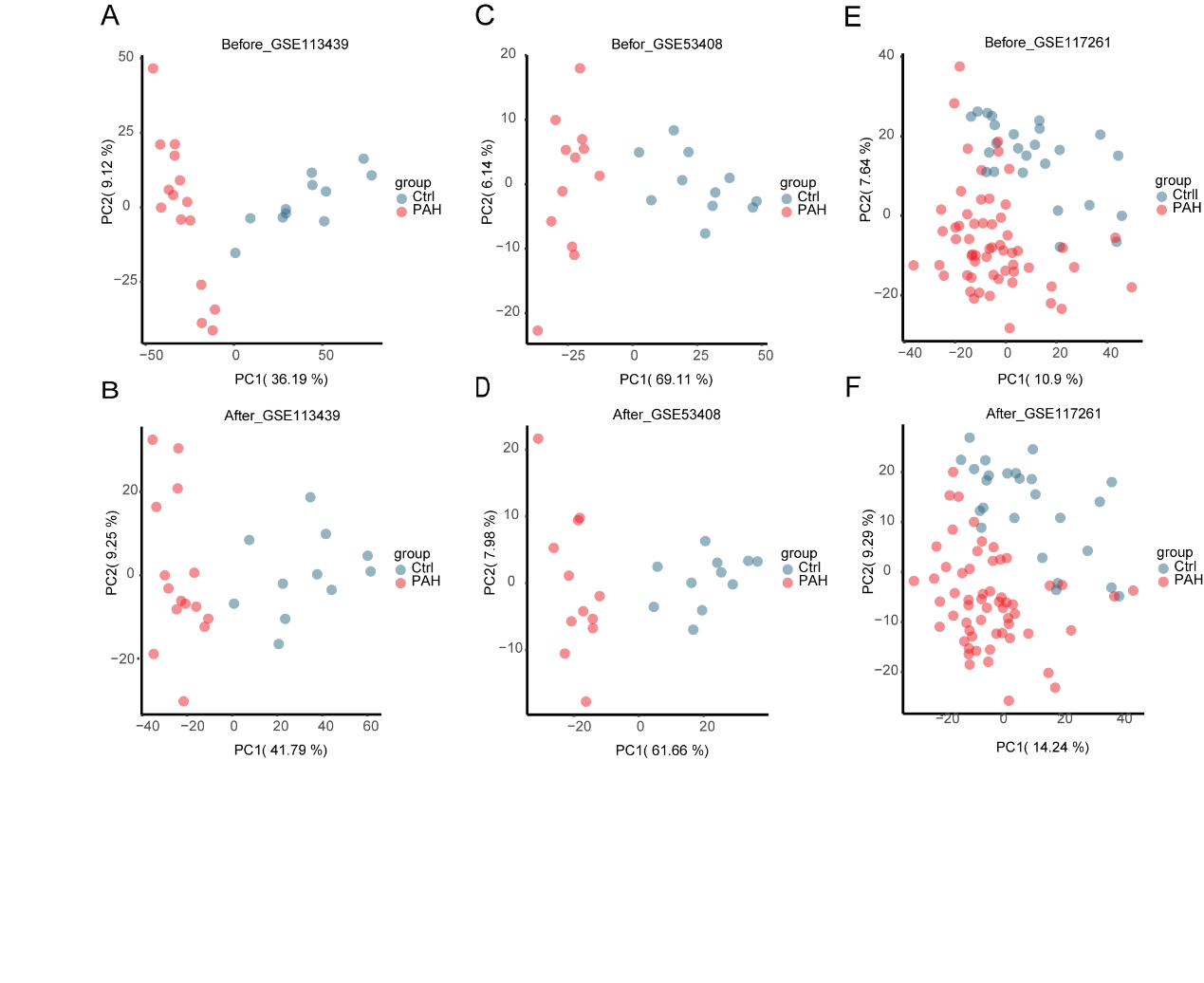


**Figure S1 PCA plots of three datasets before and after batch correction.** A-B: PCA plots of GSE113439 dataset before and after batch correction; C-D: PCA plots of GSE53408 dataset before and after batch correction; E-F: PCA plots of GSE117261 dataset before and after batch correction.


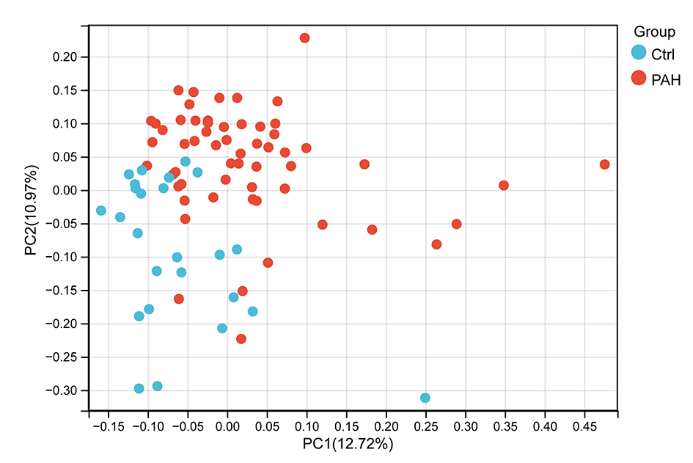


**Figure S2 The PCA plot of immune cells between PAH and control in GSE117261 dataset**


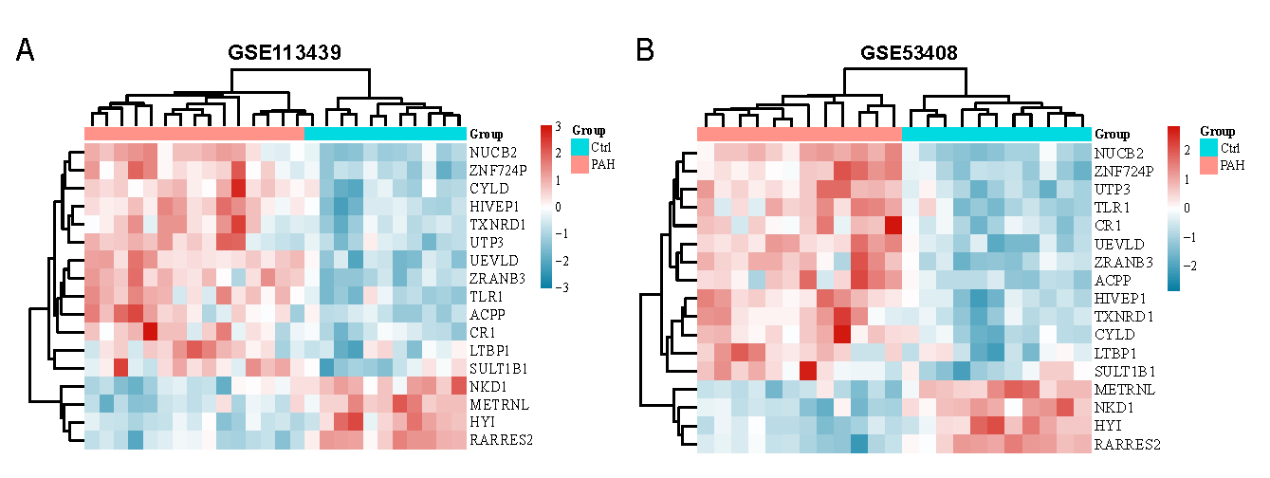


**Figure S3 Heatmap of 17 feature genes in GSE113439 and GSE53408 datasets.** A: The heatmap of 17 feature genes in GSE113439; B: The heatmap of 17 feature genes in GSE53408.


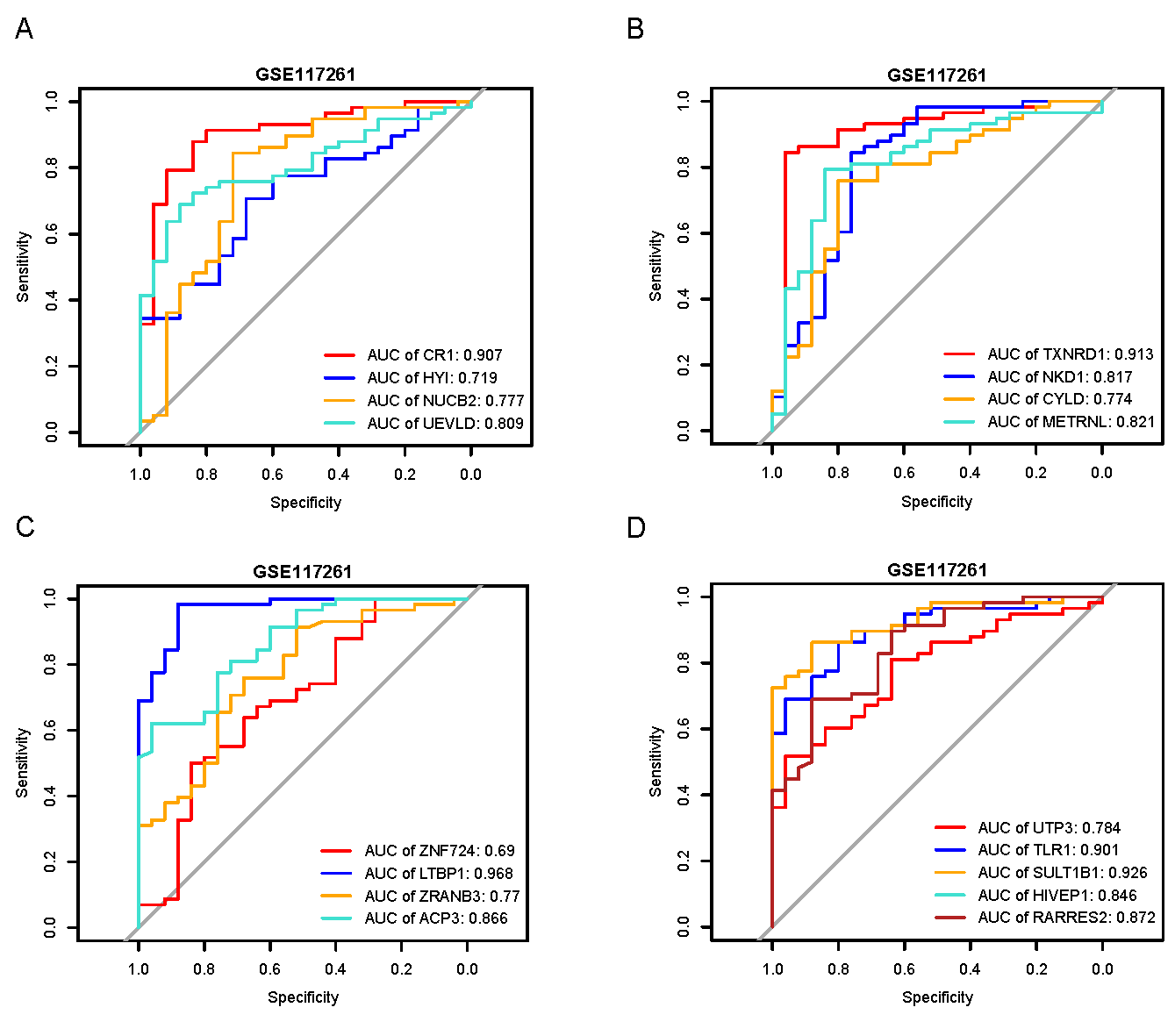


**Figure S4 The ROC of 17 genes in GSE117261.** A: ROC of CR1, HYI, NUCB2 and UEVLD; B: ROC of TXNRD1, NKD1, CCYLD and METRNL; C: ROC of ZNF724, LTBP1, ZRANB3 and ACP3; D: ROC of UTP3, TLR1, SULT1B1, HIVEP1 and RARRES2.
