# Supplementary material for: Bioinformatics analysis of the immune cell infiltration characteristics and correlation with crucial diagnostic markers in pulmonary arterial hypertension
Source: BMC Pulm Med. 2023 Aug 15;23:300. doi: 10.1186/s12890-023-02584-4 (PMC10428559; doi:10.1186/s12890-023-02584-4)
Supplement: Supplementary file 1 — Additional file 1: Figure S1. PCA plots of three datasets before and after batch correction. Figure S2. The PCA plot of immune cells between PAH and control in GSE117261 dataset. Figure S3. Heatmap of 17 feature genes in GSE113439 and GSE53408 datasets. Figure S4. The ROC of 17 genes in GSE117261. Table S1. Details of the DEGs in the dataset GSE117261. Table S2. Identification of seventeen characteristic genes of PAH using LASSO regression algorithm. Table S3. The genes in the dark olive green module by WGCNA. Table S4. The genes in the dark green module by WGCNA. [file 12890_2023_2584_MOESM1_ESM.zip › Supplementary material/Table S2.docx]

**Table S2 Identification of seventeen characteristic genes of PAH using LASSO regression algorithm**

| **Gene symbol** | **Name** |
| --- | --- |
| CR1 | complement C3b/C4b Receptor 1 |
| HYI | hydroxypyruvate isomerase (Putative) |
| NUCB2 | nucleobindin 2 |
| UEVLD | UEV and lactate/malate dehyrogenase domains |
| TXNRD1 | thioredoxin reductase 1 |
| NKD1 | NKD inhibitorof Wnt signaling pathway 1 |
| CYLD | CYLD lysine 63 deubiquitinase |
| METRNL | meteorin like, glial cell differentiation regulator |
| ZNF724 | zinc finger protein 724 |
| LTBP1 | latent transforming growth factor beta binding protein 1 |
| ZRANB3 | zinc finger RANBP2-type containing 3 |
| ACP3 | acid phosphatase 3 |
| UTP3 | UTP3 small subunit processome component |
| TLR1 | toll like receptor 1 |
| SULT1B1 | sulfotransferase family 1B member 1 |
| HIVEP1 | HIVEP zinc finger 1 |
| RARRES2 | retinoic acid receptor responder 2 |
